# Establishing the tolerability and performance of tamarind seed polysaccharide (TSP) in treating dry eye syndrome: results of a clinical study

**DOI:** 10.1186/1471-2415-7-5

**Published:** 2007-03-29

**Authors:** Maurizio Rolando, Cristiana Valente

**Affiliations:** 1 Ocular Surface Unit, Department of Neurosciences, Ophthalmology and Genetics, University of Genoa, Largo R. Benzi 10, 16132 Genoa, Italy

## Abstract

**Background:**

One of the problems arising from available preparations for dry eye syndrome is the limited residence time of products on the ocular surface. In this paper, we look at an innovative new treatment for dry eye, tamarind seed polysaccharide (TSP). TSP possesses mucomimetic, mucoadhesive and pseudoplastic properties. The 'mucin-like' molecular structure of TSP is similar to corneal and conjunctival mucin 1 (MUC1), a transmembrane glycoprotein thought to play an essential role in protecting and wetting the corneal surface and may explain its increased retention on the eye surface.

**Methods:**

The activity of TSP and hyaluronic acid (HA) in the treatment of dry eye syndrome was compared in an open-label, randomised, single-centre clinical study. Thirty patients were randomised to receive three or more applications per day of either TSP 0.5%, TSP 1% or HA 0.2% (Hyalistil™) over a period of 90 days. The primary objective of tolerability was assessed by visual analogue scale (VAS), scoring of specific symptoms and the incidence of adverse events. Secondary objectives included improvement in stability of the precorneal tear film, subjective symptoms and corneal and conjunctival staining.

**Results:**

TSP 0.5% and 1% were comparable to HA 0.2% with regard to both primary and secondary objective parameters.

TSP 1% showed benefits over HA 0.2% for the subjective symptoms; trouble blinking, ocular burning and foreign body sensation.

**Conclusion:**

This study suggests that TSP 0.5% and 1% offer at least equivalent relief to HA 0.2% for dry eye syndrome. All treatments demonstrated optimal tolerability and are suitable for frequent use in the therapy of dry eye.

TSP 1% produced promising results in terms of improvements in certain patient symptoms and suggests benefits of the TSP formulation. This study paves the way for a larger study to further establish the performance and safety of TSP compared with HA and highlights the need to expand this therapeutic agent to a wider dry eye population.

## Background

Dry eye symptoms are most commonly treated with eye drops, the major component of which is usually a viscosity-enhancing polymer. Such formulations are designed to act on the mucus and aqueous layers of the tear film, replacing lost moisture and stabilising the tear film. An issue with currently available preparations is their limited residence time on the ocular surface. In this paper, we look at an innovative new treatment for dry eye, tamarind seed polysaccharide. It is thought that the increased retention time observed with TSP on the ocular surface can be explained by similarity of the structure of TSP to transmembrane mucins, such as MUC1.

Goblet cells and lacrimal glands synthesise a spectrum of mucins that are involved in the pathophysiological events that occur at the ocular surface [1]. In the tear film, a mucus gel anchors itself and, therefore, the tear film, to the ocular surface via physicochemical interactions [2,3]. The integrity of this mucus gel, together with all the layers of the tear film, is responsible for the maintenance of normal vision and ocular comfort.

Effective distribution of the tear film across the ocular surface occurs via blinking. The healthy corneal epithelium is wettable by itself because of its ability to produce and maintain the transmembrane glycoprotein layer (MUC1). MUC1 is a membrane spanning mucin, expressed by the stratified epithelium of the conjunctiva and is believed to facilitate the spread of gel-forming mucin. Mucins possess surface activity and, in physiological concentrations, the presence of the mucin layer in the tear film converts the corneal epithelium from a hydrophobic to a hydrophilic surface so that the tear film can be spread over the cornea. If the production of mucus is reduced (for example, due to goblet cell damage, age or hormonal status), [1] mucus distribution over the preocular surface is impaired, leading to poor contact of the tear film with the eye surface and a loss of film stability [4].

**Figure 1:**
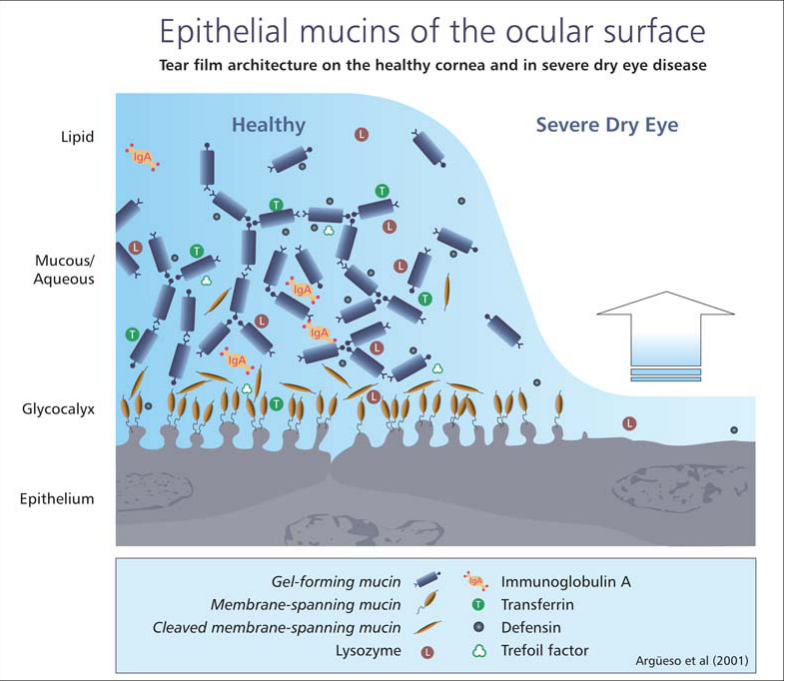
The location and extent of epithelial mucins on the ocular surface.

Figure 1 shows the location and extent of epithelial mucins on the ocular surface in a healthy eye compared with a severe dry eye. The last ten years have seen remarkable progress in understanding the structure and character of mucins [5]. Recent application of molecular techniques has demonstrated 14 human mucin genes, e.g. MUC1 and MUC5. Of these, the mucins are now classified into gel-forming or secreting, (e.g., MUC5), soluble, (e.g., MUC7), and transmembrane, (e.g., MUC1). Gel-forming mucins are responsible for the rheological properties of mucus, whereas transmembrane mucins form a dense barrier in the glycocalyx at the epithelial tear film interface. In healthy tear film, transmembrane-spanning mucins of the glycocalyx provide a negatively charged, hydrated, epithelial cell surface which supports and facilitates spreading of the hydrated tear film – a mucous gel – with its associated defence molecules. With loss of tear volume, lipid layer, glycocalyx mucins and/or gel forming mucins, dry spots develop on the eye, leading to keratinisation and loss of mucin gene expression by the epithelial cells. It is hypothesised that loss or alteration of the membrane-spanning mucins alone or in combination with MUC5AC-secreted mucin induces dry spot formation [6].

On the ocular surface, epithelial mucins serve as:

• Pre-ocular tear film stabilisers to prevent dehydration of the underlying epithelium

• A barrier against pathogen penetration

• Wetting and lubricant agents of the cornea and conjunctiva during blinking

• Promoters of adhesion between tear film layers through hydrogen bonding

Table 1 shows the characteristics of mucin-deficient dry eye.

**Table 1:** Characteristics of mucin-deficient dry eye

• Instability of tear film
• Presence of non-wetted areas on the corneal and conjunctival surfaces
• Decreased mucin production
• Altered mucin distribution
• Keratisation of the cornea and conjunctiva
• Loss of conjunctival goblet cells

### TSP formulation

**Figure 2:**
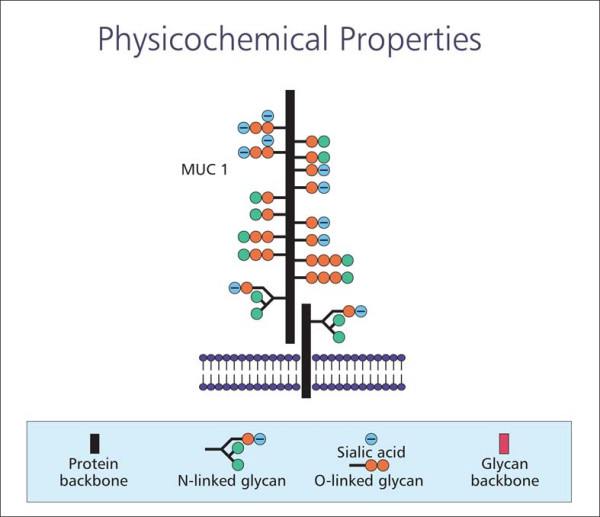
Configuration of TSP.

TSP is a new formulation derived from the tamarind seed. The main component of tamarind seed has been identified as a non-ionic, neutral, branched polysaccharide consisting of a cellulose-like backbone that carries xylose and galactoxylose substituents, [7] chemical residues similar to those of MUC1. The configuration of TSP gives the product a 'mucin-like' molecular structure, [8] with particular similarity to MUC1 (Figure 2), thus conferring optimal mucoadhesive properties.

Research has also shown that, at the concentrations present in the ophthalmic formulations studied, TSP has an important characteristic that makes it similar to natural tears, i.e. its ability to crystallise in a fern-like shape [9].

It has been suggested that the similarity of the structure of TSP to endogenous mucin may allow a formulation containing this polymer to adhere readily to the ocular surface for prolonged periods and provide sustained relief from the symptoms of dry eye [10]. Indeed, studies undertaken to date suggest that TSP may have some benefits over HA relating to ocular retention time, wound healing properties and relief of dry eye symptoms [8,11].

Overall, TSP has several physicochemical properties that make it suitable for the management of dry eye syndrome (Table 2) and which potentially have distinct advantages over currently available preparations.

**Table 2:** Physicochemical properties of TSP

• Chemical structure similar to membrane-bound ocular mucins
• Non-Newtonian rheologic behaviour
• Ferning pattern similar to natural tear film
• Mucomimetic, mucoadhesive and pseudoplastic properties

A study was performed to test this promising new agent against HA in the treatment of dry eye syndrome.

## Methods

This open-label, randomised, comparative clinical study compared the activity of TSP 0.5% and 1% vs. HA 0.2% (Hyalistil™). A total of thirty patients with dry eye syndrome were recruited (TSP 0.5% *n *= 11; TSP 1% *n *= 10; HA 0.2% *n *= 9).

### Patient demographics

Baseline characteristics of enrolled patients are summarised in Table 3.

**Table 3:** Demographic characteristics and medical history data*

	TSP 0.5% (n = 11)	TSP 1% (n = 10)	HA 0.2% (n = 9)
Gender			
Female	6 (54.5%)	6 (60.0%)	8 (88.8%)
Male	5 (45.4%)	4 (40.0%)	1 (11.1%)
Age (years)			
Mean (SD)	59.01 (13.83)	62.33 (13.06)	59.45 (10.60)
Min – Max	41.28 – 82.36	47.16 – 90.62	45.11 – 70.34
Sjogren's Syndrome	3 (27.3%)	4 (40.0%)	3 (33.3%)

* No statistically significant differences were observed between groups

Subjects were given three or more applications per day of the randomised study treatment over a period of 90 days. Patients were included if they were over 18 years of age, had a tear break-up time (BUT) < 10 seconds, dry eye symptoms (2 >6 cm on VAS), a Schirmer I test = 5 mm/5 min and positive testing = 2 in at least one area of ocular surface. Patients were excluded if they were pregnant or breastfeeding, had eye surgery in the previous three months, were on other ocular therapies or had other eye pathologies.

The primary objective of this study was to evaluate the tolerability of topical ocular administration of TSP in patients presenting with dry eye. This was assessed by a specific VAS tolerability questionnaire and by data relating to adverse events. Secondary objectives were to evaluate improvement in the stability of the precorneal tear film with the study treatments and to assess any changes in subjective symptoms and ocular surface staining.

Table 4 shows the assessment of tolerability and performance.

**Table 4:** Assessment of tolerability and performance

**Tolerability – primary objectives**
a. VAS tolerability questionnaire to evaluate:
• Blurred vision
• Ocular redness
• Ocular burning
• Ocular itching
b. Adverse events

**Performance – secondary objectives**
• Clinical/symptomatologic evaluation (symptoms were evaluated using a VAS questionnaire recording: discomfort when blinking, burning, foreign body sensation, sensation of ocular fatigue/heaviness, sensation of tearing, desire to keep eyes closed, sensation of photophobia, sensation of blurred vision and sensation of pain)
• Corneal and conjunctival staining
• Intraocular pressure (IOP)
• Tear film break-up time (BUT)
• Number of daily instillations

Tolerability data were recorded throughout the study by means of a VAS questionnaire listed per single visit. The sum total of VAS scores for all the questions, recorded after 90 days of treatment for the three groups, was evaluated by ANOVA, followed by multiple comparisons by Tukey's test. Differences relating to the trend of this variable over the 90-day study period were evaluated by means of ANOVA for repeated measurements. Any adverse events received in the study were to be described in full detail and differences amongst treatment groups evaluated accordingly (chi-square test). All treated patients were recorded in the tolerability evaluation.

Subjects in the study were assessed on days 0 (baseline), 14, 30, 60 and 90. Data have been collected from both eyes of each patients in all groups. All data have been used for statistical analysis.

At baseline, the IOP ranged from 12–19 mmHg with no difference between treatment groups and the BUT mean values were around 5 seconds for all treatments, ranging from 3–8 seconds.

This trial was carried out in accordance with the Helsinki declaration and patients' informed consent was obtained prior to commencing the study. The Study was approved by the ethics committee of S. Martino Hospital in Genoa, Italy [12].

## Results

In terms of the primary objective of evaluating tolerability, a questionnaire was used to detect the onset of blurred vision, ocular redness, ocular burning and ocular itching immediately after instillation of the preparations. For the entire duration of the study, there was no reported onset of any of the tolerability parameters assessed (Table 4). Furthermore, there were no adverse events reported throughout the study in any of the treatment groups.

Tables 5, 6, 7, 8, 9 present the results of the secondary objectives. Subjective symptoms were improved in all treatment groups (Table 5). However, there were some significant differences observed between the groups. TSP 1% showed benefits over HA in certain of the subjective VAS scores, with significant differences between treatments for the factors shown in Table 6. There were no inter-treatment differences (i.e. no superiority vs. comparator) for clinical measurements.

**Table 5:** Results

	Blinking trouble*	Ocular burning**	Sensation of foreign body***	**Sensation of lachrymation°**	**Ocular fatigue/load sensation°**	**Wish to keep eyes shut°**	**Photophobia°**
**0.5% TSP (n = 11)**							
Baseline Mean (SD)	81.55 (27.62)	86.09 (14.49)	89.64 (9.06)	8.64 (28.64)	25.45 (43.69)	31.45 (45.23)	8.82 (29.25)
Visit 5 (day 90) Mean (SD)	38.00 (22.46)	43.45 (13.92)	36.82 (15.42)	4.55 (15.08)	15.73 (27.38)	16.27 (24.69)	4.73 (15.68)
**1% TSP (n = 10)**							
Baseline Mean (SD)	81.20 (31.93)	93.00 (8.62)	90.50 (9.64)	9.50 (30.04)	9.70 (30.67)	40.60 (45.74)	14.70 (32.76)
Visit 5 (day 90) Mean (SD)	16.50 (16.21)	22.30 (13.70)	16.60 (16.79)	4.40 (13.91)	3.20 (10.12)	5.90 (10.35)	1.20 (3.79)
**0.2% HA (n = 9)**							
Baseline Mean (SD)	63.22 (40.39)	78.78 (30.76)	72.00 (31.08)	0.00 (0.00)	10.78 (32.33)	59.67 (45.17)	25.00 (38.98)
Visit 5 (day 90) Mean (SD)	40.67 (28.27)	50.44 (22.11)	42.78 (29.47)	0.00 (0.00)	9.33 (21.29)	28.67 (35.12)	12.44 (24.00)

* TSP 1% vs. HA 0.2%; p < 0.05

** TSP 1% vs. HA 0.2%; p < 0.05, TSP 1% vs. TSP 0.5%; p < 0.05

*** TSP 1% vs. HA 0.2%; p < 0.05

° p = NS

**Table 6:** Dry eye symptoms: significant inter-treatment differences

Trouble blinking	TSP 1% vs. HA 0.2%; p < 0.05
Ocular burning	TSP 1% vs. HA 0.2%; p < 0.05 TSP 1% vs. TSP 0.5%; p < 0.05
Sensation of foreign body	TSP 1% vs. HA 0.2%; p < 0.05

**Table 7:** Tear film break up time (BUT)

	TSP 0.5% (n = 11)	TSP 1% (n = 10)	HA 0.2%(n = 9)
Baseline Mean (SD)	5.18 (1.33)	5.00 (1.33)	5.22 (1.79)
Day 15 Mean (SD)	6.64 (2.11)	6.20 (1.48)	6.00 (2.18)
Day 30 Mean (SD)	7.64 (1.96)	7.20 (1.55)	6.78 (2.28)
Day 60 Mean (SD)	8.45 (2.30)	8.30 (1.42)	8.00 (2.65)
Day 90 Mean (SD)	9.64 (2.29)	9.40 (1.35)	8.44 (2.51)

**Table 8:** BUT – changes from baseline to final visit (90 days)

**Treatment**	**n**	**Mean**
0.5% TSP	11	4.45*
1% TSP	10	4.40*
0.2% HA	9	3.22*

* ANOVA between treatments; p < 0.05

**Table 9:** Summary of trial results of TSP vs. HA 0.2%

• TSP is effective at concentrations of 0.5% and 1% in treating dry eye syndrome, demonstrated by its effect on tear film break up time, corneal and conjunctival damage and its ability to provide symptom relief over a 90 day period
• TSP 0.5% and 1% show equivalent performance to HA 0.2% with regard to improving tear film break up time
• TSP 1% produced a significantly greater effect compared with HA 0.2% in some patient-scored symptoms

Furthermore, it was observed that TSP 0.5% and 1% demonstrated efficacy with significant inter-visit differences (p < 0.05) for the following:

• Subjective symptom improvements: Blinking trouble, ocular burning, sensation of foreign body, wish to keep eyes shut, photophobia, ocular pain

• Performance improvements: Tear film break up time, corneal and conjunctival damage

### Intraocular pressure

Concerning IOP, all treatments showed relatively stable values during the study period; mean values remained around 14–15 mmHg, ranging from 12–19 mmHg. Changes between baseline and day-60 visit (no assessment at final visit was foreseen) were negligible for all treatments and in both eyes.

### Average number of daily instillations

The average number of daily instillations was found to be similar in three treatment groups throughout the study, ranging between 3 and 4 instillations with no statistically significant difference.

### Tear film break up time (BUT)

In terms of tear film break up time, mean values are shown in Tables 7 and 8. Analysis of the time-course of values showed a significant increase in values between baseline and final visit (ANOVA between visits p < 0.05). There were no observed differences between treatments.

### Corneal and conjunctival staining

**Figure 3:**
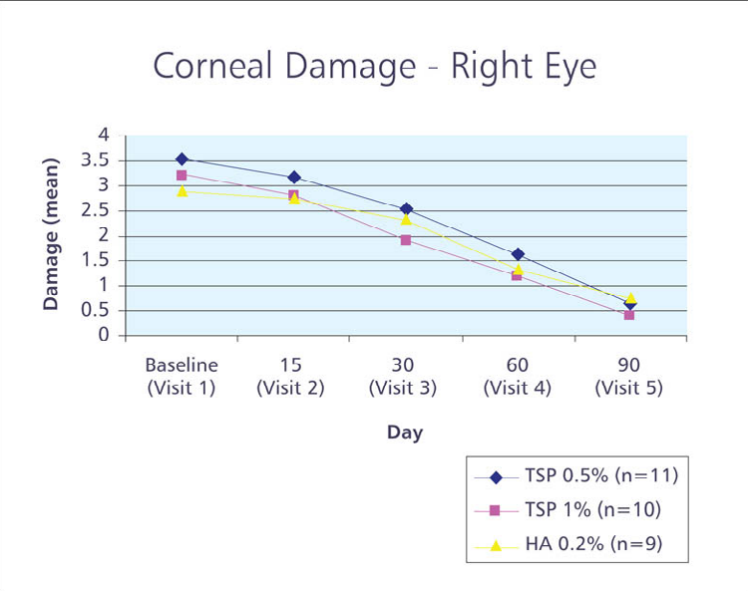
Graph showing corneal staining total score over time.

Importantly, for both corneal and conjunctival damage in both eyes, there was a statistically significant decrease in total staining score between baseline and final visit in all three treatment groups, with no statistically significant differences observed between groups (Figure 3).

## Discussion

TSP is a neutral polymer that, unlike the majority of viscosity enhancing polymers, has a branched-chain structure similar to that of corneal and conjunctival mucus transmembrane proteins. It has mucomimetic, mucoadhesive and pseudoplastic properties that may account for the benefits observed in improving dry eye signs and symptoms.

In this study comparing TSP with HA, all preparations demonstrated optimal tolerability, with no reported onset of blurred vision, ocular redness, ocular burning or ocular itching. This confirms their suitability, even for frequent use, as tear substitutes in the treatment of dry eye. Clinical performance was also demonstrated with all treatments.

All study preparations produced an improvement in many of the subjective symptoms assessed. The significant differences between products in some subjective symptom scores are interesting and warrant further investigation in a larger study population. Of particular note is the significant improvement in scores observed with TSP 1% between baseline and final visits for symptoms relating to trouble blinking, ocular burning and sensation of foreign body. These results suggest that TSP 1% may improve patient quality of life (Table 6).

The results with BUT are particularly interesting. Under normal conditions, blinking generally occurs at an average of 10–15 movements per minute (one blink every 4–6 seconds). Reports of spontaneous eye blink rate vary widely however and in some situations may be less than seven blinks per minute (one blink every 8.5 seconds). It is desirable for the tear film to remain intact between blinks so that the eye surface is 'protected' and a BUT of 8 seconds is often taken as a target. This may not only produce a benefit in terms of symptoms but also interrupts the onset of the cycle of tear instability/epithelial injury/tear instability that maintains and worsens dry eye syndrome [13]. Indeed, reports even cite that a BUT >10 seconds is required to protect ocular surface [14].

Vital dyes such as fluorescein and rose bengal are commonly used in ophthalmology to assess the extent and severity of damage to the ocular surface epithelium [15]. Importantly, statistically significant improvements between baseline and final visits were observed with respect to corneal and conjunctival staining, suggesting an improvement in the health of the ocular surface epithelium.

Lastly, the inclusion criteria used have allowed for recruitment of patients with Sjögren's syndrome. An analysis of the sub-set of patients with this condition was performed for this trial and, although patient numbers were insufficient to reach significance, trends in these data suggest an improvement in BUT and symptom scoring with TSP in this challenging patient population.

TSP clinical studies to date have produced interesting results. A randomised, blinded four-way crossover scintigraphic investigation of precorneal residence time of 3 TSP concentrations (0.5%, 1.0% and 2.0%) and HA 0.4% was conducted in 12 patients with mild to moderate dry eye syndrome, aged between 35–75 years [11]. Whilst TSP 0.5% was found to have a comparable profile to HA 0.4%, dynamic corneal residence-time curves showed that TSP 1% and 2% formulations demonstrated greater retention than HA 0.4%. The authors concluded that this pattern of retention strongly suggests a tear-structuring effect of TSP [11].

There are some limitations and additional aspects to this study worth considering. The fact that varying TSP concentrations and HA are distinguishable by appearance, viscosity and delivery device necessitates the use of an open label trial. In addition, as the trial was not placebo-controlled, patients were aware of receiving an intervention and, therefore, it is possible that this may have impacted on patients' subjective scoring of dry eye symptomatology.

## Conclusion

This study has demonstrated that TSP 0.5% and 1% are comparable to HA 0.2% according to the variables measured in the study. Due to the absence of both onset and incidence of adverse events reported throughout the study, it is concluded that all treatments demonstrated optimal tolerability and are suitable for frequent use in the therapy of dry eye. Statistically significant improvements between baseline and final visits were observed with respect to tear film break up time and corneal and conjunctival damage.

However, the results obtained with the subjective VAS symptom scores suggest benefits of the TSP 1% formulation (Table 9). It is possible that the effects seen with TSP could translate into significant differences in objective clinical measurements in a larger study population. Furthermore, data analyses indicate that TSP might, over a period of time, produce improvement in tear film stability, thereby improving eye conditions and overall patient quality of life.

## Abbreviations

HA Hyaluronic acid

TSP Tamarind seed polysaccharide

VAS Visual Analogue Scale

BUT Break up time

MUC1 conjunctival mucin 1

IOP Intraocular pressure

## Competing interests

The author(s) declare that they have no competing interests.

## Authors' contributions

RM: conceived of the study, and participated in its design and coordination, performed the study and the initial writing of the the draft manuscript.

CV: participated in the design of the study and performed the statistical analysis.

Both authors read and approved the final manuscript.

## Pre-publication history

The pre-publication history for this paper can be accessed here:


